# Hypertonic saline nasal irrigation and gargling should be considered as a treatment option for COVID-19

**DOI:** 10.7189/jogh.10.010332

**Published:** 2020-06

**Authors:** Sandeep Ramalingam, Catriona Graham, Jenny Dove, Lynn Morrice, Aziz Sheikh

**Affiliations:** 1 Department of Laboratory Medicine, Royal Infirmary of Edinburgh, Edinburgh, UK; 2 Edinburgh Clinical Research Facility, University of Edinburgh, Western General Hospital, Edinburgh, UK; 3 Centre of Medical Informatics, Usher Institute, The University of Edinburgh, Edinburgh, UK

Post-hoc secondary analysis of data from our recent Edinburgh and Lothians Viral Intervention Study (ELVIS) pilot randomised controlled trial (RCT) indicates that hypertonic saline nasal irrigation and gargling (HSNIG) reduced the duration of coronavirus upper respiratory tract infection (URTI) by an average of two-and-a-half days. As such, it may offer a potentially safe, effective and scalable intervention in those with Coronavirus Disease-19 (COVID-19) following infection with the betacoronavirus Severe Acute Respiratory Syndrome Coronavirus 2 (SARS-CoV-2) [1].

ELVIS was undertaken in 66 adults with URTI. Results have been reported in detail elsewhere [2]. Briefly, volunteers with URTI were within 48 hours of symptom onset randomised to intervention (n = 32) or control (n = 34) arms. The intervention arm made hypertonic saline at home and performed HSNIG as many times as needed (maximum of 12 times/day). Control arm participants dealt with their URTI as they normally did. Nose swabs collected at recruitment and first thing in the morning on four consecutive days were sent to the laboratory for testing. Both arms kept a diary (which included the Wisconsin Upper Respiratory Symptom Survey-21 questionnaire) for a maximum of 14 days or until they were well for two consecutive days. Follow-up data were available for 92% of individuals (intervention arm: n = 30; control arm: n = 31). HSNIG reduced the duration of URTI by 1.9 days (*P* = 0.01), over-the-counter medication use by 36% (*P* = 0.004), transmission within household contacts by 35% (*P* = 0.006) and viral shedding by ≥0.5 log_10_/d (*P* = 0.04) in the intervention arm when compared to controls [2].

We also recently reported that epithelial cells mount an antiviral effect by producing hypochlorous acid (HOCl) from chloride ions [3]. HOCl is the active ingredient in bleach. Epithelial cells have this innate antiviral immune mechanism to clear viral infections. Since bleach is effective against all virus types [4], we tested to see if a range of DNA, RNA, enveloped and non-enveloped viruses were inhibited in the presence of chloride ions supplied via salt (NaCl). All the viruses we tested were inhibited in the presence of NaCl. The human viruses we tested were: DNA/enveloped: herpes simplex virus; RNA/enveloped: human coronavirus 229E (HCoV-229E), respiratory syncytial virus, influenza A virus; and RNA/non-enveloped: coxsackievirus B3 [3].

In COVID-19, high titres of SARS-CoV-2 are detectable in the upper respiratory tract of asymptomatic and symptomatic individuals [5]. The titres are higher in the nose than the throat suggesting measures that control the infection and viral shedding will help reduce transmission [5]. In the context of the COVID-19 pandemic, we have undertaken a post-hoc re-analysis of the ELVIS data with a focus on those infected with coronaviruses. Coronaviruses were the second most common cause of URTI (after rhinoviruses). Fifteen individuals were infected by a coronavirus: 7 in the intervention arm, 8 in the control arm. In the intervention arm, four participants were infected by an alphacoronavirus (HCoV 229E = 3, HCoV NL63 = 1) and three by a betacoronavirus (HCoV HKU1 = 3). In the control arm, two were infected by an alphacoronavirus (HCoV NL63 = 2) and six by a betacoronavirus (HCoV OC43 = 1, HCoV HKU1 = 5). An individual in the control arm with HCoV HKU1 had dual infection with rhinovirus.

**Figure:**
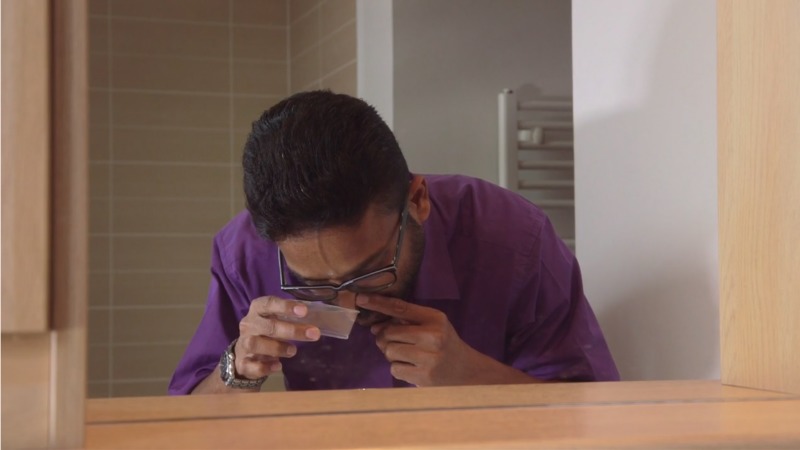
Photo: Nasal irrigation and gargling. (from the ELVIS study video, used with permission).

**Figure 1:**
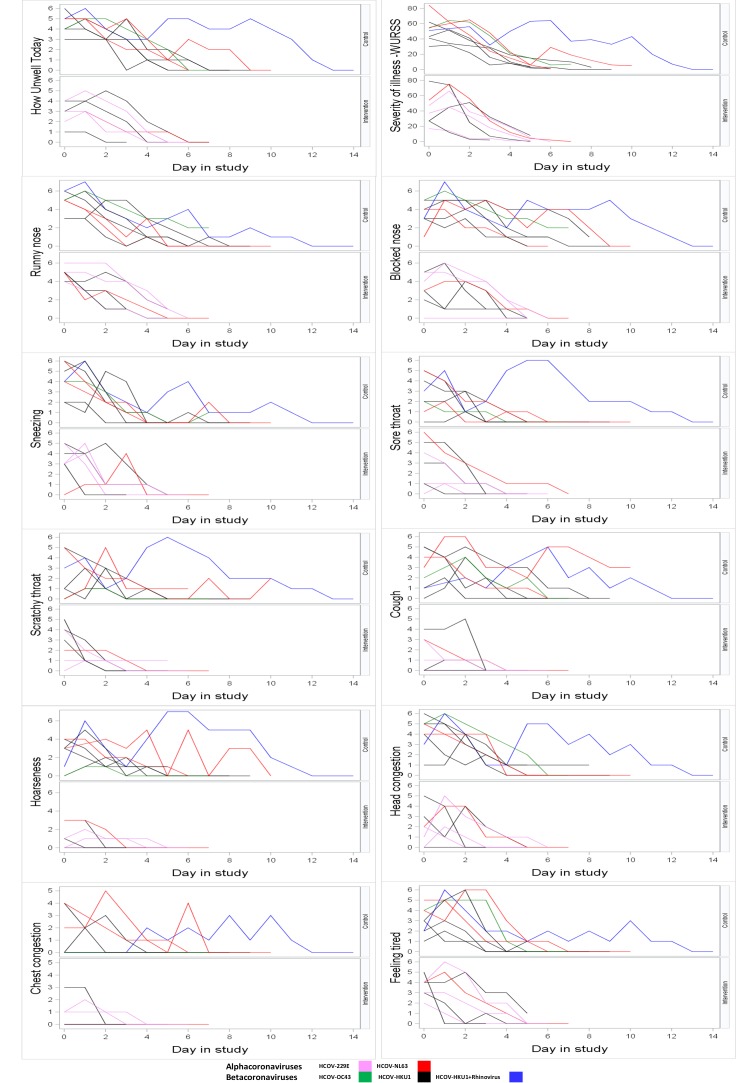
Response to global severity question and severity of symptoms. Response from participants over the study period: Each line represents response of a participant over 14 days. Data are shown by treatment group (Top panel – Control Arm; Bottom panel – Intervention Arm). The global severity question was “How unwell do you feel today”. The responses were graded from 0 (Not unwell), 1 (very mildly), 3 (mildly), 5 (moderately) and 7 (severely unwell). Likewise, each symptom was graded 0 (no symptom) to 7 (severe). WURSS-21 Score was the sum of the severity of individual symptoms.

The duration of illness was lower in the intervention arm compared to the control arm in the subset of patients infected with coronavirus (mean days (SD): 5.6 (1.4) vs 8.1 (2.9)). Using a two-sample *t* test, this was difference of -2.6 days (95% confidence interval (CI) = -5.2, 0.05; *P* = 0.054). The difference in the duration of blocked nose was -3.1 days (95% CI = -6.0, -0.2; *P* = 0.04), cough -3.3 days (95% CI = -5.9, -0.7; *P* = 0.02) and hoarseness of voice -2.9 days (95% CI = -5.6, -0.3; *P* = 0.03) in favour of HSNIG (**Table 1**). The severity of symptoms in individuals in the two arms can be seen in **Figure 1**.

**Table 1:** Number of days for self reported symptom improvement in the control and intervention arms infected by a coronavirus

Variable label	Treatment	N	Mean	SD	Difference in mean (intervention – control) (95% CI for difference)	*P*-value
Blocked nose	Intervention	7	4.0	2.2	-3.1 (-6.0, -0.2)	0.0362
Blocked nose	Control	8	7.1	2.9		
Chest congestion	Intervention	7	1.9	1.2	-0.8 (-2.7, 1.2)	0.4056
Chest congestion	Control	8	2.6	2.1		
Cough	Intervention	7	2.7	1.3	-3.3 (-5.9, -0.7)	0.0179
Cough	Control	8	6.0	3.0		
Head congestion	Intervention	7	3.4	1.9	-1.9 (-5.0, 1.1)	0.1931
Head congestion	Control	8	5.4	3.3		
Hoarseness	Intervention	7	2.4	1.6	-2.9 (-5.6, -0.3)	0.0325
Hoarseness	Control	8	5.4	2.9		
Scratchy throat	Intervention	7	2.6	1.0	-2.1 (-5.1, 1.0)	0.1712
Scratchy throat	Control	8	4.6	3.6		
Sneezing	Intervention	7	3.9	1.7	-1.0 (-3.8, 1.8)	0.4469
Sneezing	Control	8	4.9	3.0		
Sore throat	Intervention	7	3.6	1.9	-1.1 (-4.4, 2.3)	0.5139
Sore throat	Control	8	4.6	3.7		
Runny nose	Intervention	7	4.4	1.3	-1.6 (-4.1, 0.9)	0.1999
Runny nose	Control	8	6.0	2.8		
Feeling tired	Intervention	7	3.6	1.8	-2.1 (-5.1, 1.0)	0.1671
Feeling tired	Control	8	5.6	3.3		

SD – standard deviation, CI – confidence interval

The individual in the control arm with a co-existing rhinovirus infection could have affected the results. Excluding this individual, the duration of illness in the control arm was a mean of 7.3 days (SD = 1.8). The impact on the intervention control comparison was to reduce the size of the difference to -1.7 days (95% CI = -3.6, 0.2; *P* = 0.07).

In the absence of a suitable antiviral agent or a vaccine, we need a safe and effective intervention that can be globally implemented. Our *in-vitro* data gives the evidence that NaCl has an antiviral effect that works across viral types. The findings from this *post-hoc* analysis of ELVIS need to be interpreted with caution. These data do however suggest that HSNIG may have a role to play in reducing symptoms and duration of illness in COVID-19.
